# Single-nucleus transcriptomics, pharmacokinetics, and pharmacodynamics of CDK4/6 and mTOR inhibition in a Phase 0/1 trial of recurrent high-grade glioma

**DOI:** 10.1093/neuonc/noaf257

**Published:** 2025-11-08

**Authors:** Kevin C Johnson, An-Chi Tien, Jun Jiang, James McNamara, Yu-Wei Chang, Chelsea Montgomery, Anita DeSantis, Leonel Elena-Sanchez, Yoko Fujita, Seongho Kim, Avishay Spitzer, Paul Gabriel, William F Flynn, Elise T Courtois, Amy Hong, Jocelyn Harmon, Yoshie Umemura, Artak Tovmasyan, Jing Li, Shwetal Mehta, Roel G W Verhaak, Nader Sanai

**Affiliations:** Department of Neurosurgery, Yale School of Medicine, New Haven; Ivy Brain Tumor Center, Barrow Neurological Institute, Phoenix; Department of Oncology, Karmanos Cancer Institute, Wayne State University School of Medicine, Detroit (J.J., S.K., J.L.); Ivy Brain Tumor Center, Barrow Neurological Institute, Phoenix; Ivy Brain Tumor Center, Barrow Neurological Institute, Phoenix; Ivy Brain Tumor Center, Barrow Neurological Institute, Phoenix; Ivy Brain Tumor Center, Barrow Neurological Institute, Phoenix; Ivy Brain Tumor Center, Barrow Neurological Institute, Phoenix; Ivy Brain Tumor Center, Barrow Neurological Institute, Phoenix; Department of Oncology, Karmanos Cancer Institute, Wayne State University School of Medicine, Detroit (J.J., S.K., J.L.); Department of Molecular Cell Biology, Weizmann Institute of Science, Rehovot; Department of Oncology, Tel Aviv Sourasky Medical Center, Tel Aviv; Faculty of Medicine, Tel Aviv University, Tel Aviv; The Jackson Laboratory for Genomic Medicine, Farmington; The Jackson Laboratory for Genomic Medicine, Farmington; The Jackson Laboratory for Genomic Medicine, Farmington; Department of Obstetrics and Gynecology, UConn Health, Farmington; Ivy Brain Tumor Center, Barrow Neurological Institute, Phoenix; Ivy Brain Tumor Center, Barrow Neurological Institute, Phoenix; Ivy Brain Tumor Center, Barrow Neurological Institute, Phoenix; Ivy Brain Tumor Center, Barrow Neurological Institute, Phoenix; Ivy Brain Tumor Center, Barrow Neurological Institute, Phoenix; Department of Oncology, Karmanos Cancer Institute, Wayne State University School of Medicine, Detroit (J.J., S.K., J.L.); Ivy Brain Tumor Center, Barrow Neurological Institute, Phoenix; Department of Neurosurgery, Yale School of Medicine, New Haven; Ivy Brain Tumor Center, Barrow Neurological Institute, Phoenix

**Keywords:** CDK4, glioblastoma, glioma, plasticity, ribociclib, single cell, targeted therapy

## Abstract

**Background:**

Outcomes for adult patients with high-grade glioma (HGG) remain poor, necessitating new treatment strategies. Key challenges include poor drug penetration in the brain and malignant cell state plasticity. Phase 0 studies identify agents that achieve target modulation through pharmacologically relevant brain concentrations.

**Methods:**

A Phase 0/1 clinical trial combined the 2 targeted inhibitors ribociclib (CDK4/6 inhibitor) and everolimus (mTOR inhibitor) in recurrent HGG patients, aiming to identify brain-penetrant combinations and assess their impact on malignant cell states. We enrolled 24 patients with recurrent HGG, characterized by *CDKN2A*/*B* deletion or *CDK4*/*6* amplification, *PTEN* loss or *PIK3CA* mutations, and wildtype retinoblastoma protein (Rb). Tumors were evaluated for pharmacokinetics, pharmacodynamics, and single nucleus transcriptomics.

**Results:**

Median unbound ribociclib concentrations in gadolinium non-enhancing tumor regions were significantly above the biochemical IC_50_ for CDK4/6 inhibition at 400 and 600 mg QD doses. Unbound everolimus concentrations were undetectable (<0.1 nM) in tumor regions across all dose levels. Ribociclib treatment was associated with significantly decreased Ki-67-positive cells. Single-nucleus RNA sequencing of 17 on-trial IDH-wildtype recurrences and 88 standard-of-care-treated recurrences showed a significantly lower fraction of cycling and neural progenitor-like malignant cell populations in ribociclib-everolimus-treated tumors. CDK4/6 inhibitor-directed malignant cell state shifts were validated using 3 patient-derived cell lines.

**Conclusions:**

This trial underscores the value of integrating pharmacokinetics, pharmacodynamics, and single-nucleus transcriptomics in Phase 0/1 surgical studies to assess treatment effects, including malignant cell state shifts. Clini­calTrials.gov identifier: NCT03834740.

Key Points

- Ribociclib, but not everolimus, demonstrates brain penetrance at pharmacologically relevant levels in gadolinium non-enhancing regions of high-grade glioma patients.
- Recurrent IDH-wildtype glioblastomas receiving presurgical ribociclib demonstrated lower abundance of neural progenitor cell-like and proliferating malignant cells when compared with standard-of-care.
- In vitro studies of CDK4/6 inhibition confirmed a decrease in neural progenitor-like and cycling populations toward more differentiated malignant cell states.

Importance of the Study

This study provides critical insights into the pharmacokinetics and pharmacodynamics of ribociclib and everolimus in recurrent high-grade glioma enrolled in a Phase 0/1 trial. Single-cell sequencing data demonstrate that targeting CDK4, a key driver of a glioma cell state, with brain-penetrant CDK4/6 inhibitor (ribociclib) results in a smaller neural ­progenitor cell-like population in gliomas. In contrast, everolimus exhibited inadequate brain penetration, reinforcing the challenge of delivering effective mTOR inhibition to gliomas. These findings underscore the significance of Phase 0 trials in rapidly assessing drug target engagement and optimizing treatment strategies. Our work lays the foundation for future targeted inhibitor trials that aim to modulate cell state shifts for therapeutic benefit.

Standard-of-care treatment of adult patients with glioblastoma consists of maximal extent of resection followed by concomitant chemoradiation, resulting in a median overall survival of 15 months.^1^ The minimal progress that has been achieved in improving treatment outcomes suggests that innovative approaches that diverge from conventional oncological strategies are needed.^2^ Here, we present a dual-drug Phase 0 “trigger” trial that leverages pharmacokinetics (PK), pharmacodynamics (PD), and single-nucleus transcriptome sequencing to enable the discovery of treatment-associated biological signals.^3^

Focal amplifications in *CDK4*/*CDK6* and *CDKN2A* homozygous deletion in glioma represent genomic alterations that disrupt normal functioning of the cell cycle.^4^^,^^5^ Ribociclib is a highly specific small-molecule inhibitor of CDK4/CDK6,^6^ and has been shown to significantly prolong survival outcomes for patients with advanced breast cancer.^7^ We previously showed that ribociclib was well tolerated and exhibited good central nervous system (CNS) penetration in a Phase 0/1 clinical trial of patients with high-grade glioma (HGG).^8^ Analysis of tissues from patients who experienced a recurrence while on ribociclib monotherapy treatment suggested an increase in the mammalian target of rapamycin (mTOR) pathway activity, highlighting a potential mechanism of resistance. Combined CDK4/6 and mTOR inhibition (everolimus) was further supported by preclinical data that indicates synergy against glioblastoma.^9^ These results prompted initiation of a Phase 0/1 trial with ribociclib and an mTOR inhibitor, everolimus, described here. In addition to tumor PK and PD analysis, in-depth molecular analyses of tumor specimens from the Phase 0 trial represent an opportunity to quickly and broadly understand mechanisms of action and malignant cellular plasticity in response to investigational drugs.

Single-cell and nucleus RNA sequencing studies have previously shown that most glioblastomas consist of malignant cell states that include stem-like populations of neural progenitor cell-like (NPC-like) and oligodendrocyte progenitor-like (OPC-like) cells as well as more differentiated populations of astrocyte-like (AC-like) and mesenchymal-like (MES-like) cells, with subsets of malignant cells in each cell state additionally being characterized by an active cell cycle signature.^10–13^ The distributions of malignant cell states have been shown to be influenced by both the microenvironment and genetics, including an association between greater NPC-like signal and *CDK4* amplifications.^11^ We have previously shown that direct application of stressors (eg, hypoxia) results in cell state shifts,^13^ and that acquired genetic alterations can lead to consistent changes in cell states.^14^^,^^15^ The drivers of such transitional shifts may be multifactorial and include therapy or underlying genetic evolution. Profiling human glioma samples that are actively on targeted drugs (eg, CDK4/6 inhibitors) is needed to understand how these therapies influence tumor cell biology and demonstrate whether it is possible to pharmacologically shift malignant cellular states.

In this current Phase 0/1 trial, we investigated the combination of ribociclib (CDK4/CDK6 inhibitor) plus everolimus (mTOR inhibitor) in recurrent HGG to enhance therapeutic response. This study was designed to determine the primary outcome measures of (1) the total and unbound concentrations of both ribociclib and everolimus in contrast-enhancing and non-enhancing tumor tissue, (2) identify the molecular effects of both drugs via assessment of proximal pharmacodynamic markers (eg, pRb, pS6, and p4EBP1) and single-nucleus transcriptomic analyses, and (3) determine the maximum tolerated dosing regimen for the drug combinations. The trial was designed to qualify patients for a Phase 1 expansion if a patient’s tumor samples met pharmacokinetic and pharmacodynamic thresholds for both therapies. Overall, our study highlights the importance of integrating PK/PD and posttreatment molecular characterization to assess glioma treatment effects from experimental therapeutics.^3^

## Methods

### Sample Acquisition and Study Design

**Figure 1.**
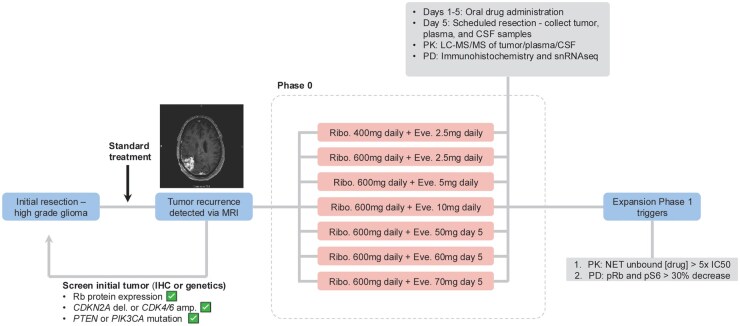
Phase 0/1 study design for ribociclib and everolimus in recurrent high-grade glioma. An overview of the clinical trial design and sample collection. Patients with a tumor recurrence detected via magnetic resonance imaging (MRI) had their prior tumor screened for immunohistochemistry (IHC) and/or targeted genetic sequencing to select patients most likely to demonstrate a response to CDK4/6 and mTOR inhibition. As part of the Phase 0 design, patients were allocated to 7 possible treatment arms including 400 or 600 mg ribociclib (Ribo.) daily for 5 days with dual inhibition of everolimus (Eve.) ranging from 2.5 to 10 mg daily for 5 days or a single higher dose on day 5 of treatment at 50-70 mg. Following the fifth day of drug exposure, the patients underwent a scheduled tumor resection. Tumor (gadolinium contrast-enhancing and non-enhancing regions), cerebrospinal fluid (CSF), and plasma specimens were assessed with a combination of pharmacokinetics (PK; tumor, plasma, and CSF) using liquid chromatography with tandem mass spectrometry and pharmacodynamics (PD; tumor) using IHC and single nucleus (sn) RNA sequencing. For patients to be eligible for the Phase 1 expansion, the patient’s tumor tissue needed to meet pharmacokinetic/pharmacodynamic criteria for both ribociclib and everolimus. NET = non-enhancing tumor tissue.

This Phase 0/1 clinical trial (NCT03834740) was conducted at the Ivy Brain Tumor Center at the Barrow Neurological Institute/St Joseph’s Hospital and Medical Center. Patient enrollment spanned from 2019 to 2022. Enrolled Phase 0 patients (inclusion criteria: Supplementary Methods) were administered 400-600 mg once daily (QD) of ribociclib and 2.5 mg QD to 70 mg once weekly (QW) of everolimus for 5 days preceding planned cranial tumor resection (Figure 1). Preoperative MRI and intraoperative neuronavigation were used to collect tumor specimens from contrast-enhancing and non-enhancing regions. Blood plasma and cerebrospinal fluid (CSF) were also collected from patients at the time of surgical resections. These specimens were used for PK and PD analyses. Resected tumor tissue for 21 specimens was immediately frozen to process for single-nucleus RNA (snRNA) sequencing. In 2 cases, patients that received ribociclib-everolimus therapy (5 days) were later deemed to have presented with treatment-associated pseudo-progression following surgical resection. These patients were replaced on the trial for pharmacokinetic and pharmacodynamic analyses; however, these tumor samples were profiled with snRNA following the drug treatment and resection. One pseudo-progression snRNA sample was excluded due to low malignant cell count, while the other was retained due to malignant cell detection. Among the 24 tumors, 21 were confirmed to be IDH-wildtype, while 3 were high-grade IDH-mutant astrocytoma. IDH-wildtype tumors were used in all subsequent analyses. IDH-mutant tumor data are included in statistical analyses only where indicated.

### Ribociclib and Everolimus Pharmacokinetics

Pharmacokinetic evaluation was conducted as previously described^8^ using a validated liquid chromatography with tandem mass spectrometry (LC-MS/MS) method.^16^ Briefly, blood samples were collected, and plasma was separated from whole blood via centrifugation (4°C, 1500 × *g* for 10 minutes). Tumor samples collected from contrast-enhancing/non-enhancing regions were rinsed with cold PBS to remove any residual blood, dried, and snap frozen. CSF samples were intraoperatively collected.

### Ribociclib and Everolimus Pharmacodynamics

Pharmacodynamic biomarkers for ribociclib were assessed using immunohistochemistry (IHC) with the following antibodies: anti-pRb (Cell Signaling Technology; #8516, 1:400), anti-pFOXM1 (Cell Signaling Technology; #14655, 1:200), anti-Ki-67 (DAKO; M724029, 1:100); and anti-cleaved caspase 3 (Cell Signaling Technology; #9661, 1:300). Everolimus pharmacodynamic biomarkers were assessed using IHC with the following antibodies: anti-p4EBP1 (Cell Signaling Technology; #2855, 1:500); anti-pS6 ribosomal protein (Cell Signaling ­Technology; #4858, 1:100). IHC staining was performed using the BOND RX automated system (Leica Biosystems) as previously described.^8^ Stained slides were imaged on a Leica Versa microscope and analyzed with Aperio Image software. Percentage of stain-positive cells was quantified from at least 6 randomly selected regions of interest within the tumor as assessed by a board-certified neuropathologist.

### Single Nucleus RNA Sequencing Tumor Samples

Available frozen clinical glioma specimens were obtained from patients that had received combination therapy of ribociclib and everolimus (*n *= 21 samples) or ribociclib monotherapy (*n* = 3 samples). Frozen glioma specimens (*n* = 3) from a ribociclib monotherapy trial (NCT02933736) were analyzed to expand the dataset of samples treated with ribociclib.^8^ Nuclei isolation for all samples was performed within the same laboratory using an assay optimized for the isolation of nuclei from non-diseased brain and brain tumor tissues (protocols.io: dx.doi.org/10.17504/protocols.io.81wgb657ylpk/v1).

### Single Nucleus RNA Sequencing Analysis

Cellranger count (6.1.2) function was used to align sequencing reads from FASTQ files to the GRCh38-2020-A reference transcriptome with the parameter include introns set to true. Count matrices were loaded into R using Seurat’s (version 4.3.0) Read10X function. Cells were filtered based on the following quality control criteria: number of genes detected greater than 500 and fewer than 10 000; maximum percentage of mitochondrial genes of 5%. DoubletFinder was used to identify potential nuclei doublets, which were filtered out.^17^ The expression data were processed by normalization, scaling, PCA based on genes differentially expressed across brain cell types, Harmony batch correction for sample preparation batch,^18^ Uniform Manifold Approximation and Projection (UMAP), and clustering using the Louvain algorithm, and resolution set to 0.6. Cell type annotation was based on gene expression markers for each cluster and confirmed via mapping to a normal reference brain atlas (Azimuth). InferCNV (https://github.com/broadinstitute/inferCNV) was used to confirm malignant status based on the presence of copy number alterations. Predicted copy number altered regions based on a moving average of a 101-gene window were determined with the Hidden Markov Model (HMM) parameter enabled. HMM-predicted copy number alteration levels were extracted to define malignant and nonmalignant cells based on the following approach. Cells assigned to a cluster consistent with malignant marker expression that also possessed greater than 25% of chromosome 7 inferred gain and/or 25% of chromosome 10 inferred loss (2 hallmark alterations in HGG^4^^,^^5^^,^^19^) were retained as malignant. Putative malignant cells not meeting this threshold were labelled as unresolved and excluded. Cells assigned to a nonmalignant gene expression cluster with 25% of chromosome 7 inferred gain and/or 25% of chromosome 10 inferred loss were labelled as unresolved and excluded. Samples with fewer than 25 malignant cells were excluded from malignant state analysis. Seurat’s AddModuleScore function was used to score malignant cells separately within each tumor sample for the relative expression of each Neftel cell state (AC-like, MES-like, OPC-like, NPC-like) and cell cycle.^11^ Cell state assignment was determined based on Neftel cell state with the highest relative expression. Cell cycle status was assigned independent of Neftel cell state. Cells were classified as AC-like, etc., along with its cell cycle status—cycling or not.

### Publicly Available Single Nucleus RNA Sequencing Data Cohorts

Raw gene expression counts from single nucleus RNA sequencing (snRNAseq) (10x Genomics) for longitudinally collected primary and recurrent glioblastoma samples were retrieved from GSE274546 (*n* = 59 patients)^14^^,^^20^ and GSE174554 (*n* = 31 patients).^10^ Notably, only recurrent IDH-wildtype samples were analyzed for differences with IDH-wildtype ribociclib-everolimus-treated cohort.

### Determination of Ribociclib Dose In vitro

Patient-derived glioma stem cell lines (GSCs; GB126, GB86, and GB239) were established from resected GBM tumor tissue. Cell lines were genetically profiled for mutations and copy number aberrations using the IvySeq custom gene panel. All cell lines harbored high-level *CDK4* amplifications among other alterations. GB239 was derived from a tumor sample included in this Phase 0 trial (tumor sample: 100-0039). All human GSCs were cultured as described previously.^12^ GB126, GB86, and GB239 were treated with 0.5, 1, and 5 μM concentrations of ribociclib and incubated for 5 days to determine ribociclib dose per cell line. The dose of ribociclib (0.5-5 μM) used per cell line was empirically determined as the dose at which phosphorylated Rb protein expression was reduced by 50%.

### Ribociclib-Treated In vitro Single-Cell RNA Sequencing and Analysis

The ribociclib and DMSO control experiments were performed in a passage-controlled fashion such that cells were split from the same parental line and then were allocated to either receive ribociclib or DMSO for a duration of 5 days. Following the fifth day of drug exposure, cells were harvested, cryopreserved to be processed at a later point, or immediately processed for single-cell RNA sequencing (10x Genomics, 3’ version 3). The single-cell RNA sequencing data were then processed via cellranger count and Seurat. Briefly, cells were filtered out based on the following criteria: minimum number of genes detected >500 and <10 000 with a maximum percentage of mitochondrial genes set to 10%. Statistical assessment was made between the malignant proportions of each cell state using a 2-sided paired *t*-test.

### Statistical Methods

Descriptive statistics of the clinical data are included in Table 1. Comparisons between groups were conducted using a 2-sided or paired 2-sided *t*-test, where indicated. *P* < .05 was considered significant. Multiple hypothesis test corrections (ie, false discovery rate correction) were applied for snRNAseq pseudobulk differential expression analysis.

**Table 1.** Clinical Characteristics for Ribociclib-Everolimus Phase 0 Clinical Trial Cohort

Characteristic	Phase 0 (*n* = 24)
	Recurrent tumors
**Age at recurrent resection (years)**	
Median (LQ-UQ)	58 (46-65)
Unknown (*n*)	0
**Sex (%)**	
Male	17 (71%)
Female	7 (29%)
**IDH mutation status (%)**	
IDH-wildtype	21 (88%)
IDH-mutant	3 (12%)
**Ribociclib dose (QD—5 days)**	
400 mg	6 (25%)
600 mg	18 (75%)
**Everolimus dose**	
2.5 mg QD**—**5 days	9 (37.5%)
5 mg QD**—**5 days	3 (12.5%)
10 mg QD**—**5 days	3 (12.5%)
50 mg once on day 5	3 (12.5%)
60 mg once on day 5	3 (12.5%)
70 mg once on day 5	3 (12.5%)

## Results

### Patient Population

Patient demographics and clinical characteristics are summarized in Table 1 (CONSORT diagram; Supplementary Figure S1). Eligibility was determined based on previous clinical records, panel DNA sequencing, and IHC of archival tissue from a previous surgery (Figure 1, Supplementary Figure S2A). There were 21 patients with an IDH-wildtype GBM and 3 patients with a high-grade IDH-mutant astrocytoma enrolled (Table 1). All patients received either 400 or 600 mg/day of ribociclib for 5 days prior to a scheduled brain tumor resection. Six patients received 5 days of ribociclib (400 mg QD) plus everolimus (2.5 mg QD) and underwent tumor resection at 2, 8, or 24 hours following the last dose. Six dose-escalation cohorts (*n* = 3 each) reached a maximum dose level of ribociclib (600 mg QD) plus everolimus (70 mg QW, Figure 1). The dose levels and clinical outcomes for patients in this cohort are presented (Supplementary Table S1, Supplementary Figure S2B). Both drugs were administered orally, and the combination was overall well tolerated. No grade 4 or 5 adverse events related to study treatment were observed. A single serious adverse event (grade 2; pancytopenia) was noted in one patient on dose level 5. All treatment-related adverse events are listed in Supplementary Table S2. Since no patients qualified for the Phase 1 trial portion, neither clinical safety nor efficacy could be assessed.

### CNS Pharmacokinetics and Pharmacodynamics

To evaluate tumor PK and PD, samples were collected at the time of surgical resection, which followed 5 days of combined ribociclib and everolimus exposure. The following samples were collected for pharmacokinetic evaluation: tumor specimens from the gadolinium-enhancing tumor region, tumor specimens from non-enhancing tumor region, plasma, and CSF. Total and unbound drug concentrations for ribociclib and everolimus in tumor, plasma, and CSF samples were determined using a validated LC-MS/MS method and equilibrium dialysis, as previously described.^16^ Measuring unbound drug concentrations in the non-enhancing tumor regions is critical to assess drug penetration as non-enhancing tumor regions possess an intact blood-brain barrier (BBB), are often unresectable, and are source of radiotherapy-induced neurological morbidity.^21^

**Figure 2.**
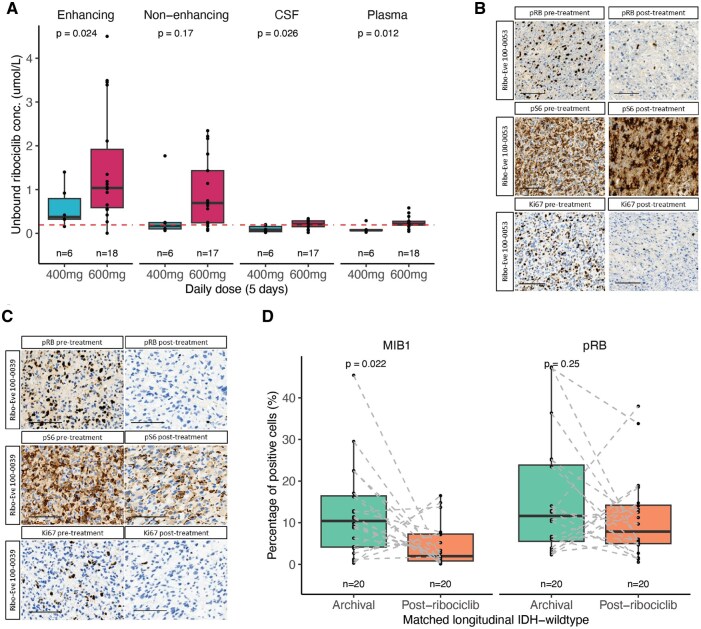
Pharmacokinetic and pharmacodynamic analyses of tumor samples exposed to ribociclib. (A) Unbound ribociclib concentrations in the gadolinium-enhancing tumor, non-enhancing tumor, cerebrospinal fluid (CSF), and plasma samples are presented for daily doses of 400 and 600 mg. Box plots in this figure and throughout the manuscript represent the median, lower and upper quartiles, and the whiskers indicate 1.5 times the interquartile range. Each point represents an individual measurement. Statistical differences were assessed with 2-sided *t*-test. (B, C) Representative IHC images for pre-treatment (ie, archival surgical sample) and posttreatment (Phase 0 ribociclib exposure) for key pharmacodynamic markers in 2 subjects are shown. Scale bar, 100 microns. (D) Box plots represent the percentage of positive cells for each pharmacodynamic marker of ribociclib response including Ki-67 and phosphorylated Rb (*n* = 20 IDH-wildtype). The dotted lines represent connections between samples collected from the same patient at the archival time point and following ribociclib Phase 0 therapy. Statistical differences were assessed with a paired 2-sided *t*-test.

As previously seen in our monotherapy trial, there was robust CNS penetration of ribociclib as illustrated by total and unbound drug concentrations in tumor regions and CSF (Supplementary Figure S3A, Figure 2A).^8^ This was further supported by tumor- and CSF-to-plasma partition ratios for the total (Kp) and unbound drug (Kp, uu, Supplementary Figure S3B and C). Following 5 days of exposure at the daily oral dose of 400 and 600 mg, ribociclib achieved a median unbound drug concentration of 170 nM (range, 65-1770 nM) and 693 nM (range, 68-2345 nM) in the non-enhancing tumor regions, respectively (Supplementary Table S3). These data were consistent with the unbound ribociclib concentrations in the non-enhancing tumor regions (median, 482 nM; range, 327-1295 nM) observed in ribociclib monotherapy study where the patients received a 5-day exposure at the daily dose of 900 mg.^8^ Notably, we observed a significant dose-dependent increase in unbound ribociclib concentration in the contrast-enhancing region and CSF (*t*-test, *P* = .02 and *P* = .03, Figure 2A) and a trend toward increased concentration in the non-enhancing region (*t*-test, *P *= .17, Figure 2A), suggesting that the higher dose can deliver increased intratumoral concentration without dose-associated toxicities. The extent of ribociclib penetration in non-enhancing regions as assessed by Kp, uu was consistent across the different dose levels, with the median Kp, uu of 3.19, 3.78, and 2.00 at the daily dose of 400, 600, and 900 mg (monotherapy trial), respectively (Supplementary Table S3, Supplementary Figure S3C). Additional total and unbound ribociclib pharmacokinetic parameters are presented per individual in Supplementary Table S4. Pairwise comparisons of enhancing and non-enhancing regions from the same tumors highlighted the increased ribociclib concentration in enhancing regions and the importance of assessing non-enhancing unbound drug concentrations (Supplementary Figure S3D, 2-sided paired *t*-test, *P *= .035).

CNS penetration of everolimus was evaluated at the daily oral dose of 2.5, 5, and 10 mg (given daily for 5 days), as well as at a single oral dose of 50, 60, and 70 mg (given on day 5). Overall, total everolimus concentrations and Kp indicated CNS penetration across different dose levels (Supplementary Figure S4A and B, Supplementary Table S5). The median everolimus CSF concentration was 0.48 nM (range, below limit of quantification—11.4.5 nM) across the different dose levels, indicating a weak drug CNS penetration (Supplementary Figure S4A and B, Supplementary Table S5). The total everolimus pharmacokinetic parameters are reported per individual in Supplementary Table S6. The unbound drug concentrations in both enhancing and non-enhancing tumor regions were below the lower limit of quantitation (<0.1 nM), suggesting that inadequate levels of the pharmacologically active, unbound form of everolimus reach the tumor tissue in HGGs.

To assess the impact of these drugs on their targets, we next investigated the longitudinal stability of previously identified pharmacodynamic biomarkers of ribociclib response and activation of the mTOR pathway.^8^ Given the tumor type differences based on IDH status and small number of IDH-mutant samples (*n* = 3), we first restricted the longitudinal pharmacodynamic analysis to IDH-wildtype tumors. For IDH-wildtype patients with available pharmacodynamic data (*n* = 20), we observed a significant decrease in the proliferative Ki-67 marker (paired *t*-test*, P *= .02) but a nonsignificant decrease in phosphorylated Rb, which is a downstream target of CDK4/6 (paired *t*-test*, P *= .25, Figure 2B-D, Supplementary Table S7), for the surgical samples treated with ribociclib compared with their matched archival tissue samples. The reduction in Ki-67 remained consistent when IDH-mutant tumors were included in the analysis (paired *t*-test *P *= .04, Supplementary Figure S5A) and when stratifying by different genetic backgrounds (Supplementary Figure S5B and C). While we observed a consistent decrease in Ki-67, we cannot exclude the possibility that differences in the tumor microenvironment also contributed to these changes. The reduction in Ki-67 was not statistically significant between tumors treated with the two doses of ribociclib (median decrease 8.94% 600 mg versus 2.93% 400 mg, *t*-test *P *= .34, Supplementary Figure S5E). Pathway activity of mTOR, as quantified by p4EBP1 and pS6, was elevated but not significantly different at the surgical specimen compared to archival tissue suggesting variability in the upregulation of the mTOR pathway following CDK4/6 inhibition (paired *t*-test, *P *> .05, Supplementary Figure S5A).

The requirements for expansion from the Phase 0 treatment (5 days of treatment preceding surgical resection) to Phase 1 were that both ribociclib and everolimus unbound drug concentrations needed to be 5 times greater than the biochemical IC_50_ (40 nM for ribociclib, 2 nM for everolimus) and a greater than 30% decrease in both longitudinal pRb and pS6 levels (Figure 1). Accordingly, none of the Phase 0 patients qualified for Phase 1 portion of the study.

### Single Nucleus RNA Sequencing of Phase 0 Tumor Samples

**Figure 3.**
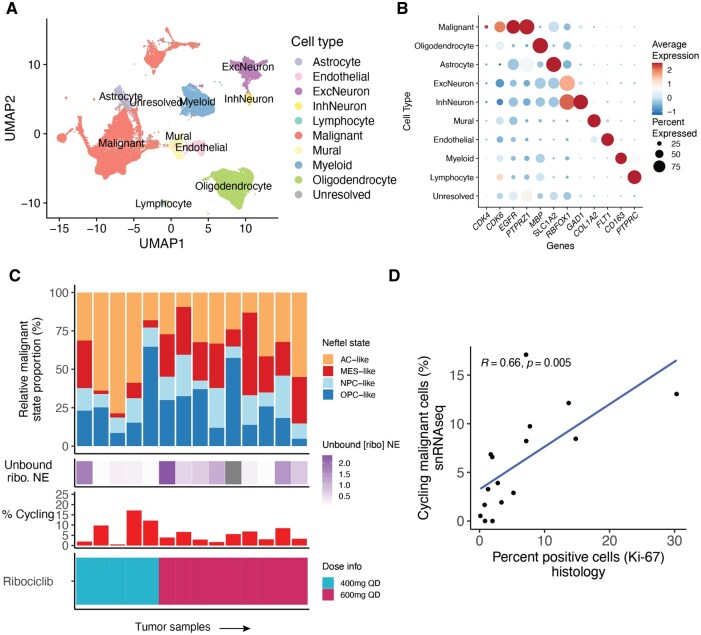
Single nucleus RNA sequencing (snRNAseq) of tumor samples treated with dual ribociclib and everolimus inhibition. (A) UMAP visualization for gene expression-based clusters that correspond to major cell types (*n* = 87 888 cells, *n* = 24 samples including IDH-wildtype *n* = 22 and IDH-mutant *n* = 2). Each point represents a single cell, and the colors denote cell type. Cell types were identified via integrated approach that considered de novo clustering identification, mapping to a nonmalignant reference brain atlas, and inferred copy number alterations. (B) Dot plot represents the average normalized gene expression data per cell type for key marker genes. Dot color reflects expression scale and dot size reflects the percentage of cells annotated as a cell type expressing the displayed genes. Genes shown represent selected genes from Seurat’s FindMarkers for each cell type as well as *CDK4*/*CDK6* expression. (C) Stacked bar plots represent the relative malignant proportions assigned to each Neftel cell state in the top panel (*n* = 14 IDH-wildtype samples from Phase 0 ribociclib-everolimus cohort with snRNA and PK data). The middle top panel reflects the unbound ribociclib concentration measured in the contrast non-enhancing region of the tumor. The middle bottom panel represents the proportion of malignant cells that are actively cycling according to snRNA. The bottom panel represents the daily ribociclib oral dose (5 days preceding resection). (D) For samples with sufficient snRNA cells passing quality control and IHC, the Pearson correlation between histological estimates of cycling cells and cycling malignant cells in the snRNA data is presented (*n* = 16, IDH-wildtype + IDH-mutant).

To better understand the cellular changes that may occur in tumors due to active ribociclib exposure, we profiled 21 tumor samples with snRNAseq including: 19 IDH-wildtype samples and 2 IDH-mutant samples. In addition, we also profiled 3 IDH-wildtype samples collected from a ribociclib monotherapy trial (NCT02933736), where 2 samples were longitudinally collected from the same patient.^8^ There were 87 888 cells remaining for analysis following application of quality control filters (Supplementary Figure S6A and B). Unsupervised clustering followed by UMAP was used to identify the major tumor microenvironment cell types including neurons, oligodendrocytes, astrocytes, myeloid, lymphocytes, endothelial cells, mural cells, and malignant cells (Figure 3A, Supplementary Figure S6C and D). These cell type identities were confirmed by the high expression of key cell type-specific marker genes, including high expression of *CDK4*/*CDK6* in a subset of malignant cells (Figure 3B). We confirmed malignant cell status by inferring large-scale copy number alterations and identified 41 422 malignant cells (Methods, median 1132 malignant cells per tumor, Supplementary Figure S7A and B). We restricted subsequent analyses to tumors with a minimum of 25 malignant cells (Supplementary Figure S7C). We next assigned malignant cell state by scoring each cell within the tumor for the IDH-wildtype gene expression metaprograms previously identified in Neftel et al.(IDH-wildtype tumors only, Figure 3C)^11^ assigning a malignant state based on highest relative metaprogram expression. We also determined whether a cell was actively in the cell cycle independent of the Neftel cell states by scoring for cell cycle metaprogram expression (Methods). To determine how snRNAseq metrics of cycling cells compared with IHC-based proliferation markers, we correlated the proportion of malignant cells that were actively cycling in the snRNAseq data with the percent of Ki-67 positive cells from matched IHC samples. We observed that these 2 metrics of proliferation were positively correlated with one another (Pearson’s correlation coefficient = 0.66, *P *= .005, Figure 3D), providing confidence in the snRNA cycling status assignment.

### Malignant Cell State Shifts Are Associated With Ribociclib Exposure

Next, to understand ribociclib’s impact on intratumoral malignant cell state diversity, we sought to compare the relative malignant cell states with 2 independent cohorts of recurrent IDH-wildtype tumors that received the standard-of-care therapy (ie, the alkylating agent temozolomide, radiation, and surgery) but were not exposed to neoadjuvant ribociclib prior to the recurrent tumor resection.^10^^,^^14^  Supplementary Table S8 shows a comparison of the standard treatment and Phase 0 cohorts based on subject demographics. Overall, 57 (standard treatment—Cellular Analysis of Resistance and Evolution [CARE] cohort) and 31 (standard treatment—Wang et al.cohort) recurrent GBMs that received standard therapy without neoadjuvant ribociclib exposure were analyzed for snRNAseq.

**Figure 4.**
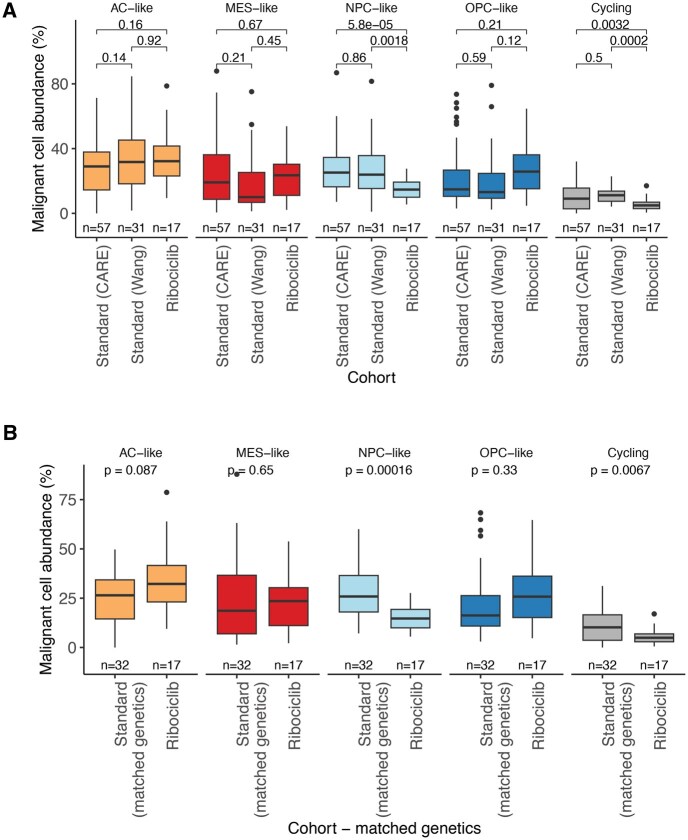
Malignant cell state distribution differences in standard treatment versus neoadjuvant ribociclib following standard treatment cohorts. (A) The relative malignant cell proportion is presented for the samples treated with standard of care therapy (*n* = 57 snRNA IDH-wildtype recurrences in the CARE cohort, *n* = 31 snRNA IDH-wildtype recurrences from Wang et al. and samples treated with neoadjuvant ribociclib that followed previous standard therapies (*n* = 17 IDH-wildtype recurrences). The 4 malignant cell states (AC-like, MES-like, NPC-like, OPC-like) plus the proportion of malignant cells that are actively cycling are shown. Box plot spans from the first to third quartiles, median values are indicated by a horizontal line, whiskers show 1.5× interquartile range, and outlier sample points are shown. Statistical difference was assessed with 2-sided *t*-test. (B) A subset of the CARE IDH-wildtype recurrences where genetic profiles matched the inclusion criteria from the ribociclib-everolimus cohort (*n* = 32 IDH-wildtype recurrences). Statistical difference was assessed with 2-sided *t*-test.

We first assessed whether there were any shifts in malignant cellular state distributions between the standard treatment cohorts and ribociclib cohort. We observed that a lower proportion of malignant cells that were actively cycling (2-sided *t*-test, *P *= 3.2E-03 and *P *= 2.0E-04) in those exposed to ribociclib providing orthogonal support to the observation that ribociclib suppresses cell proliferation in vivo (Figure 4A). We also found a decrease in the neural progenitor-like (NPC-like) state for the ribociclib-exposed samples (2-sided *t*-test, *P* = 5.8E-05, *P* = 1.8E-03, Figure 4A). Notably, there were no significant differences in malignant state abundance between the 2 standard cohorts. For the standard treatment CARE cohort where genetic information was available, we restricted to recurrent tumors that harbored molecular alterations that matched genetic inclusion criteria (eg, *CDKN2A* loss, *CDK4* amp) for the current trial and observed consistent significant shifts in cellular states for both cycling (*P* = 6.7E-03, Figure 4B) and NPC-like cells (*P* = 1.6E-04, Figure 4B). Separately, we applied an independent glioblastoma cell state classifier based on gene expression pathway activity,^22^ and observed that the proliferative, progenitor population was less abundant in the ribociclib cohort samples with a shift toward a metabolic state linked with longer survival (Supplementary Figure S8A). The NPC-like state has been previously associated with *CDK4* amplifications, and our results suggest that inhibitors of CDK4 selectively downregulates the NPC-like and similar expression programs.^11^ Among the ribociclib monotherapy samples, we longitudinally profiled a patient’s tumor from both a Phase 0 (5-day drug exposure) and Phase 2 (104 days) using snRNAseq. While only a single case, we did observe a reduced fraction of NPC-like malignant cells in the Phase 2 sample (Supplementary Figure S8B).

We next assessed whether there were any differences in the tumor microenvironment (TME) abundance across cohorts since the TME plays a role in shaping malignant cell state abundance.^11^ We observed significantly greater oligodendrocyte abundance in the standard treatment cohorts versus ribociclib-exposed cohort but not in the abundance of malignant or myeloid cells (Supplementary Figure S8C). Importantly, we confirmed that NPC-like malignant state abundance was not associated with TME cell type abundance, suggesting that the observed cohort difference in NPC-like abundance was not likely driven by cohort differences in the local microenvironment (Supplementary Figure S8D). Together, these comparative analyses across cohorts support the anti-proliferative effect of ribociclib in IDH-wildtype gliomas and suggest that ribociclib exposure may be associated with a smaller NPC-like malignant state compartment.

### In vitro Ribociclib Exposure Depletes Cycling and NPC-like Cell States

**Figure 5.**
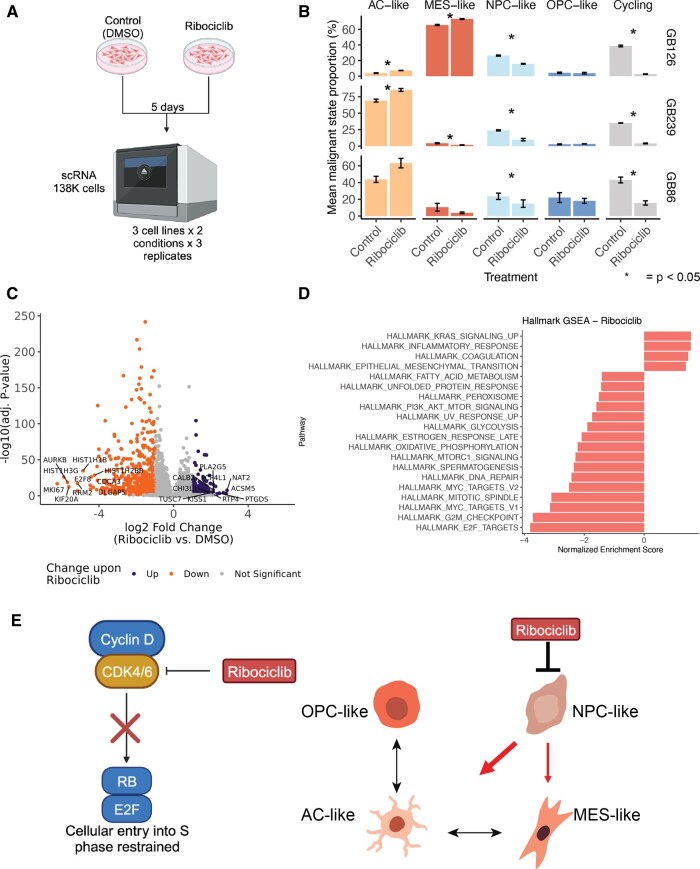
In vitro pharmacological perturbation of malignant cell states. (A) Experimental design for ribociclib in vitro perturbation experiments. Patient-derived cells were split and allocated to either control (DMSO) or ribociclib treatment for 5 days at empirically defined doses that inhibit pRb1 to 50% per cell line. Cells were then profiled by single-cell RNA sequencing. Each experiment was performed with 3 biological replicates. (B) The relative proportions of each malignant state are shown per cell line. The height indicates the mean value and the bars +/− the standard error. Statistical assessment was made with a paired 2-sided *t*-test. Asterisk (*) indicates comparisons below the *P* < .05 threshold. (C) Volcano plot represents the pseudobulk differential gene expression analysis. Each point reflects an individual gene with point color reflecting upregulation following ribociclib exposure (purple) and downregulation (orange) based on absolute(log2 FC) > 1 and adjusted *P*-value < .05. Y-axis indicates the −log10(adjusted *P*-value). The top 10 up- and down-regulated gene names are shown. (D) Gene set enrichment analysis (GSEA) for differentially expressed genes between ribociclib exposure and DMSO. Bars show normalized enrichment score, where values less than 0 would reflect underrepresentation in the differential expression rank (eg, E2F targets downregulated in ribociclib exposure). (E) Ribociclib’s impact on cellular states in HGG. Ribociclib penetrates glioma tissues to inhibit CDK4/6 activity leading to hypo-phosphorylation of Rb and restrained entry into the cell cycle (left panel). Ribociclib exposure shifts malignant states away from NPC-like populations to more differentiated cell states (eg, AC-like and MES-like, right panel). *Created in BioRender. Chowdhury, T. (2026)*. https://BioRender.com/vowektp and https://BioRender.com/fh4hz6p.

To confirm whether ribociclib exposure selectively depletes or enriches for a particular malignant state, we exposed patient-derived recurrent glioblastoma cell lines to ribociclib or vehicle control (DMSO) in vitro (Figure 5A). These patient-derived cell lines (GB126, GB239, and GB86) were selected based on similar genomic profiles to the eligibility criteria (eg, all had a high-level *CDK4* amplification, Supplementary Figure S9A). To establish the selected ribociclib dose for each cell line, we exposed cells to ribociclib for 5 days across a dose range and selected the dose per cell line that achieved a 50% inhibition of pRb (Methods, Supplementary Figure S9B, dose 0.5-5 µM). We next performed single-cell RNAseq on the ribociclib-treated (*n* = 3 per cell line) and DMSO control samples (*n* = 3 per cell line). Following filtering for quality cells (138 870 cells post-quality control, *n* = 18 samples), we performed dimensionality reduction and clustering (Supplementary Figure S9C). This revealed cell line-dependent and treatment-enriched clusters suggesting a treatment effect (Supplementary Figure S9D). We found that *CDK4* gene expression was reduced but remained high following ribociclib treatment, consistent with ribociclib binding to CDK4 to inhibit its function (Supplementary Figure S9E).

We then scored the cells for malignant state gene expression metaprograms (Neftel states) and examined their relative proportions across treatment groups (Methods, Figure 5B). Across all 3 cell lines, we found a consistent decrease in the cycling cells (paired *t*-test*, P *= 8.7E-4 GB126, *P *= 5.4E-4 GB239, *P *= .04 GB86) consistent with the cytostatic effect due to cell cycle arrest.^23^ The relative NPC-like malignant state proportion was also reduced for all 3 cell lines (paired *t*-test*, P *= 4.3E-4 GB126, *P *= .02 GB239, *P *= .038 GB86) with a compensatory increase toward AC-like (paired *t*-test*, P *= 6.6E-3 GB126, *P *= .03 GB239, *P *= .06 GB86) and in one cell line the MES-like state (GB126), both of which represent more differentiated-like malignant cells (Figure 5B).^13^ These cell state changes were not accompanied by changes in broad inferred chromosomal gains or losses events (Supplementary Figure S10A). Additionally, when we restricted our analyses to those cells not actively cycling, we observed a consistent decrease in NPC-like (paired *t*-test, *P *= 1.4E-06) and increase in AC-like (paired *t*-test, *P *= 2.6E-03), supporting this pharmacological driven cell state shift can occur independent of cell cycle (Supplementary Figure S10B).

To identify broader patterns of ribociclib activity, we performed pseudobulk differential gene expression analyses where cells from a sample are collapsed into a single gene expression profile to perform cell state independent analyses. This analysis tested the effect of treatment while adjusting for experimental replicate and identified 678 upregulated and 582 downregulated genes (adj. *P* < .05, abs(log2FC) > 1, Supplemental Table S9) in the ribociclib-treated samples (Figure 5C). A gene set enrichment analysis on significantly downregulated genes in the ribociclib-treated cells identified cell cycle as the most affected hallmark pathway (Figure 5D). In contrast, the upregulated genes were enriched for programs including inflammatory response and epithelial-to-mesenchymal transition nominating candidate programs that may drive resistance to ribociclib. Together with our Phase 0 in vivo results, these in vitro findings highlight ribociclib’s ability to modify glioma cellular state distribution via cell cycle downregulation and a shift away from the NPC-like state to more differentiated malignant cell states (Figure 5E).

## Discussion

In this dual-drug Phase 0 trial with a pharmacokinetic/pharmacodynamic-triggered Phase 1 expansion, we provide a template for an innovative trial design that allows dose escalation and rapid, early-stage evaluation of PK, PD, and molecular impacts of 2 targeted agents, ribociclib and everolimus. Importantly, this design incorporated single-cell-level transcriptional analyses to comprehensively characterize tumor samples on active therapy. Beyond confirmation of effective ribociclib penetration and ineffective everolimus penetration into both contrast-enhancing and non-enhancing tumor regions, our study provides critical insights into ribociclib’s ability to exert significant anti-proliferative effects on glioma cells, as evidenced by reduced cell proliferation markers and shifts in malignant cell states towards more differentiated phenotypes. Our study also suggests that a higher ribociclib dose may achieve a greater biological effect. In contrast, everolimus’ lack of CNS penetration raises concern about pursuing its further use in adult glioblastoma therapy. Together, our combined in vivo and in vitro results provide proof-of-concept that a drug that therapeutically targets malignant cell states can penetrate glioma tumor tissue and have a biological effect.

The integration of snRNA-seq technology into the Phase 0 study design provided an unbiased insight into the impact of targeted therapies on different cellular states within the tumor milieu. While pharmacodynamic analysis of changes in expression of proximal markers provides information on target inhibition to some extent, snRNA-seq analysis provides broader information on overall cell state and cell cycle regulation. For example, our analyses identified molecular effects of ribociclib on cell cycle regulation as evidenced by decreased proliferating malignant and NPC-like populations, among which the latter could not have been detected by traditional pharmacodynamic analyses.^11^ Our observations of smaller cycling and NPC-like populations were confirmed and extended in our in vitro perturbation analyses, where the shift away from the NPC-like cell state was coupled with a concomitant increase in more differentiated malignant states (AC-like and MES-like). The ability to pharmacologically induce a differentiation effect in glioma cells was recently shown in IDH-mutant tumors that were treated as part of a Phase 1 clinical trial with a mutant IDH inhibitor, examined with snRNAseq, and compared with publicly available IDH-mutant single-cell reference profiles.^24^ Together, the mutant IDH inhibitor trial and our CDK4/6 inhibitor trial’s results highlight the utility of using single-cell genomics to profile glioma tissue samples on trial to understand how drugs function in vivo. Beyond transcriptional profiling, complementary molecular modalities (eg, proteomics) may be needed to assess other therapeutic classes such as protein degraders. More broadly, these studies also suggest that pharmacologically shifting malignant cells towards a more differentiated malignant state may restrict glioma cell plasticity by depleting progenitor populations and slow tumor growth via cell cycle inhibition.^25^

Although CDK4/6 or mTOR inhibitors have not been effective as monotherapies in glioblastoma, the frequent dysregulation of these 2 pathways suggests potential utility as a combination therapy.^8^^,^^26^ While ribociclib showed robust penetration and a biological effect, our study detected no unbound everolimus in the glioma tissue across 6 dose escalation levels. These findings indicate that everolimus may have limited clinical potential in adult glioblastoma treatment. However, they highlight the need for further investigation into its PK and PD properties, particularly in relation to its established therapeutic role in subependymal giant cell astrocytoma in patients with tuberous sclerosis.^27^ Given the importance of the mTOR signaling pathway in glioma pathogenesis, alternate strategies are needed to modulate the mTOR pathway more effectively within the glioma microenvironment.

The study design used in this trial allowed investigation of 2 targeted therapies concomitantly with dose de-escalation and escalation rules to accommodate challenges with drug toxicities and unsuitability of microdosing due to BBB. However, there were some study design limitations. Unlike conventional Phase 0 studies in the medical oncology field, the comparator for pharmacodynamic response in Phase 0-collected tissue was archival tissues from a previous surgery and not pretreatment biopsies. Collecting adequate pretreatment glioma tumor tissue required for molecular analyses such as IHC and snRNA remains a challenge in evaluating glioma patient treatment response, where additional biopsies may pose an increased risk to the patient.^3^^,^^28^

In summary, our Phase 0/1 trial of ribociclib and everolimus offers valuable insights into the therapeutic potential of CDK4/6 inhibition for HGGs. The insights gained from single-cell analyses have provided a deeper understanding of ribociclib’s impact on malignant cell states, offering a promising avenue for future therapeutic interventions. As the neuro-oncology field moves forward, the integration of innovative trial designs, molecular profiling techniques, and non-invasive longitudinal sampling methods will be pivotal in accelerating the development of therapies for glioma that improve patient outcomes.

## Supplementary Material

noaf257_Supplementary_Data

## Data Availability

All analyses were performed in R 4.2.0. Gene expression count matrices processed by cellranger have been deposited to ­Synapse (https://www.synapse.org/Synapse:syn60087246/wiki/628483).
