# Supplementary material for: Single-nucleus transcriptomics, pharmacokinetics, and pharmacodynamics of CDK4/6 and mTOR inhibition in a Phase 0/1 trial of recurrent high-grade glioma
Source: Neuro Oncol. 2025 Nov 8;28(3):659–71. doi: 10.1093/neuonc/noaf257 (PMC13070491; doi:10.1093/neuonc/noaf257)
Supplement: noaf257_Supplementary_Data [file noaf257_supplementary_data.zip › noaf257_Supplementary_Data/RiboEve_NeuroOncology_SupplementaryMethods_20250924.docx]

**Supplementary Methods**

**Study Inclusion Criteria**

1. Patients needed to be 18 years or older.
2. Prior resection of histologically grade III or IV glioma (2016 World Health Organization diagnostic criteria) that progressed following standard therapy (i.e., Stupp regimen of maximally safe surgical resection, temozolomide, and fractionated radiotherapy).
3. The tumor recurrence needed to be confirmed with Gadolinium contrast-enhanced magnetic resonance imaging (MRI) or diagnostic biopsy followed by pathologic review.
4. Archival tissue needed to demonstrate one of the following: a) Rb protein positivity on immunohistochemistry (>= 20%) or the absence of *RB1* mutations; b) chromosomal loss of *CDKN2A/B/C* or *CDK4/6* or *CCND1/2* amplification on microarray-based comparative genomic hybridization; c) mTOR positive with *PTEN* loss or *AKT3* amplification or mutations for *PIK3CA* or *PIK3R1*, or pS6 positivity (>= 10%).
5. Patients needed to voluntarily agree to participate by signing a written informed consent document.
6. Patients needed to be able to swallow ribociclib and everolimus capsules/tablets.

**Western Blotting**

Cellular protein from cultured cells were homogenized in RIPA lysis buffer containing protease and phosphatase inhibitors (ThermoFisher Scientific), rotated at 4 ̊C for 20 minutes and then centrifuged at 15,000 rpm for 10 minutes at 4 ̊C. Protein concentration from whole-cell extracts were determined using the Bradford Protein Assay (ThermoFisher Scientific). Equal amounts of protein (10-40 μg/lane) were loaded onto a 10% or 12.5% SDS- PAGE gels and transferred to a polyvinylidene fluoride membrane (PVDF; Millipore-Sigma).

Membranes were blocked with 5% non-fat milk for 1 hour at room temperature and incubated overnight with primary antibody at 4 ̊C. Primary antibodies used in this study were anti-pRb (Cell Signaling Technology; #8516, 1:1000), anti-total RB (Cell Signaling Technology; #9309, 1:2000) and mouse anti- β-actin (1:1000, Bio-Rad, MCA5775GA). Membranes were probed with fluorophore-conjugated anti- mouse or anti-rabbit secondary antibodies (1:10,000; ThermoFisher Scientific). Western blots were developed using the LI-COR Odyssey CLx imaging system (LI-COR Inc.) and quantitated using the Image Studio Lite software.
