# Supplementary figures and images for: Single-nucleus transcriptomics, pharmacokinetics, and pharmacodynamics of CDK4/6 and mTOR inhibition in a Phase 0/1 trial of recurrent high-grade glioma
Source: Neuro Oncol. 2025 Nov 8;28(3):659–71. doi: 10.1093/neuonc/noaf257 (PMC13070491; doi:10.1093/neuonc/noaf257)

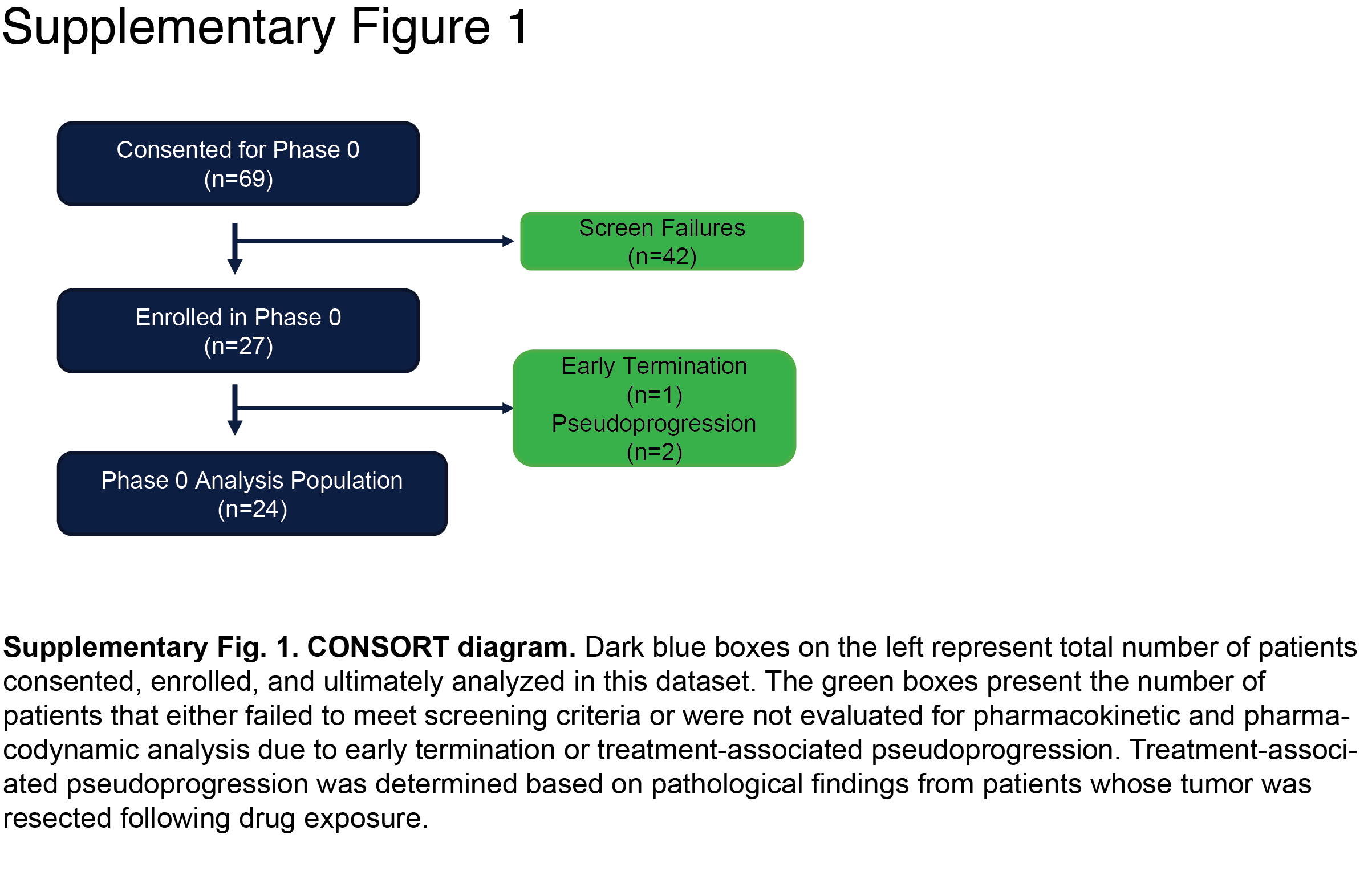

Supplement: noaf257_Supplementary_Data [file noaf257_supplementary_data.zip › noaf257_Supplementary_Data/SupplementaryFigure1.png]

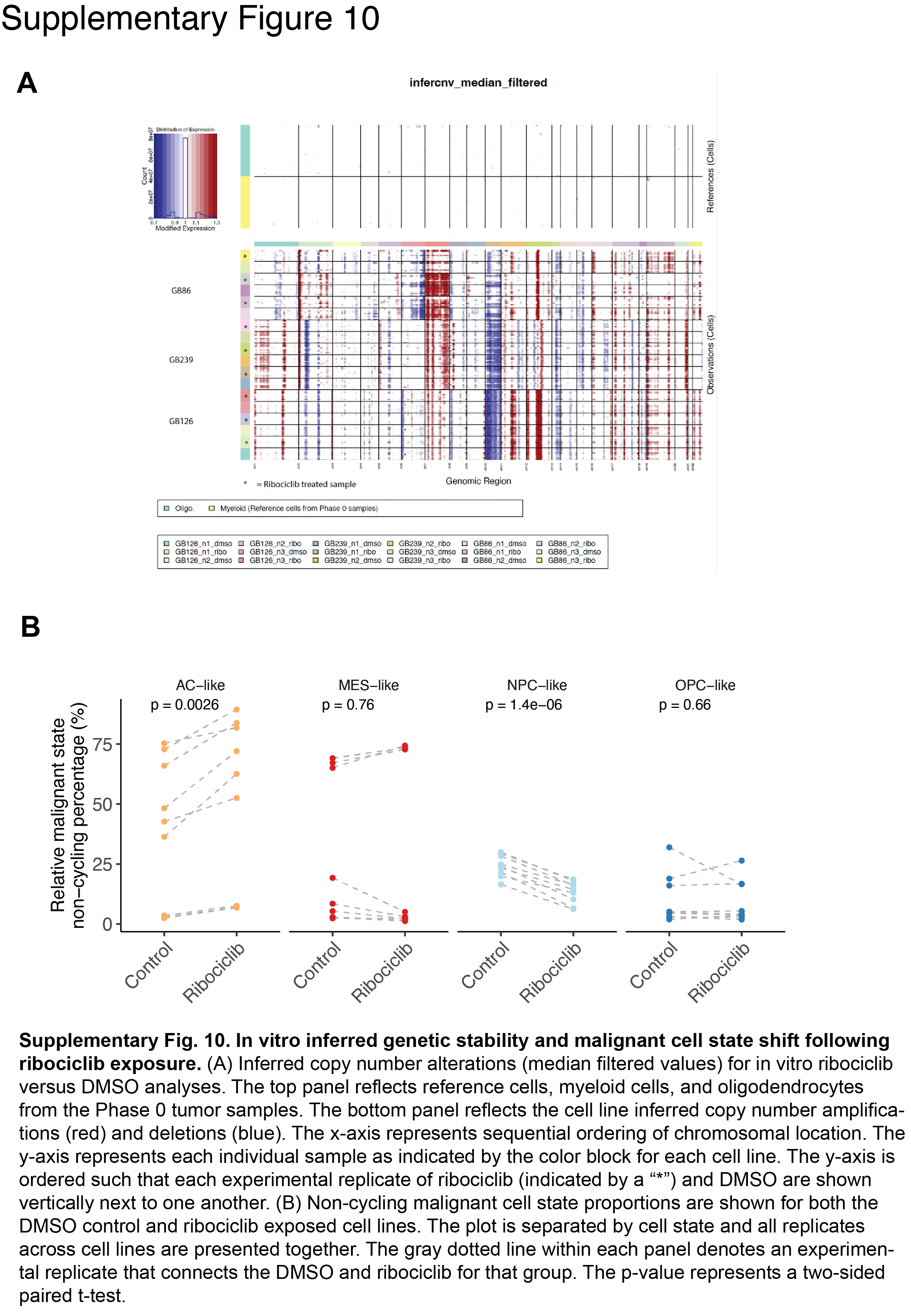

Supplement: noaf257_Supplementary_Data [file noaf257_supplementary_data.zip › noaf257_Supplementary_Data/SupplementaryFigure10.png]

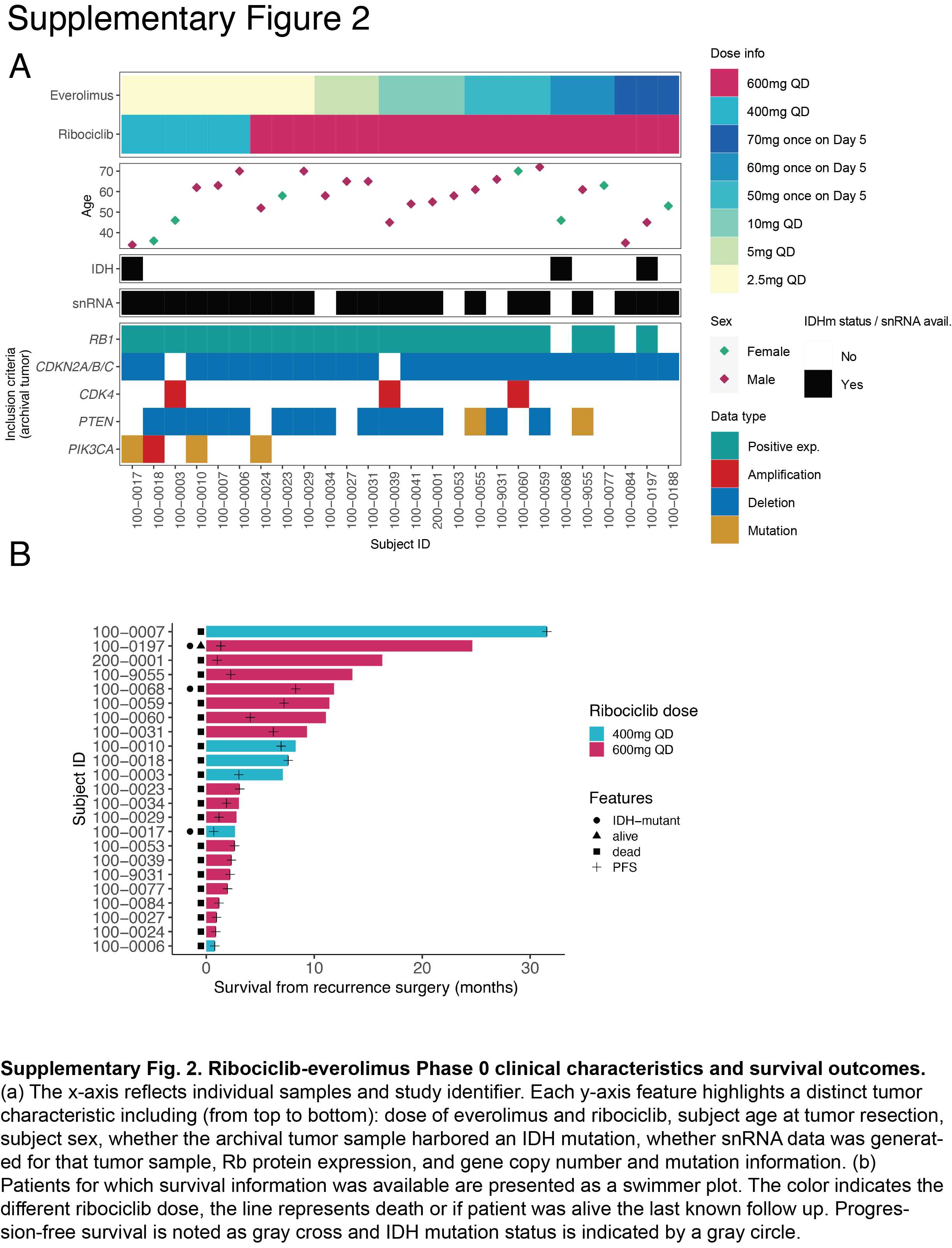

Supplement: noaf257_Supplementary_Data [file noaf257_supplementary_data.zip › noaf257_Supplementary_Data/SupplementaryFigure2.png]

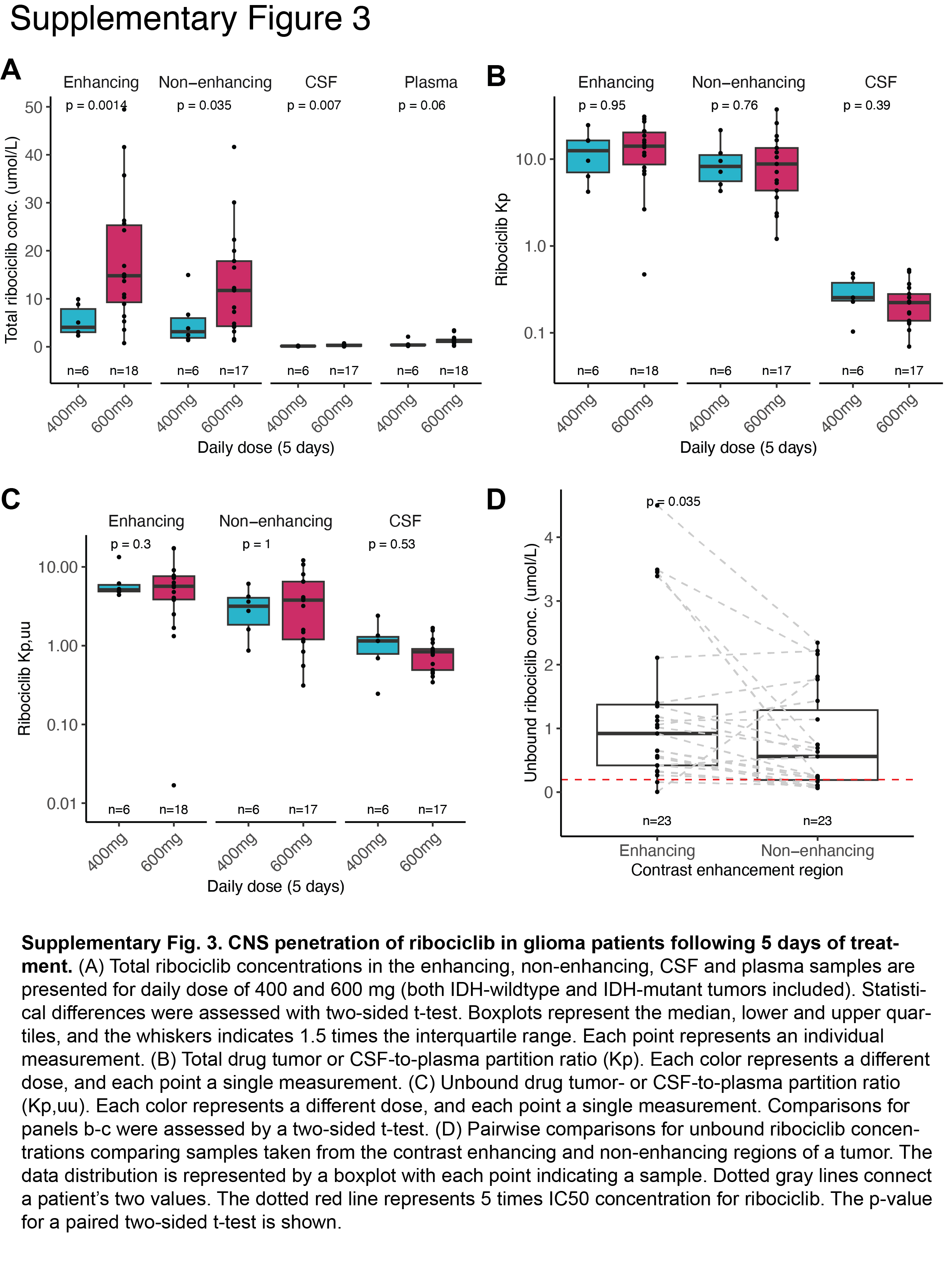

Supplement: noaf257_Supplementary_Data [file noaf257_supplementary_data.zip › noaf257_Supplementary_Data/SupplementaryFigure3.png]

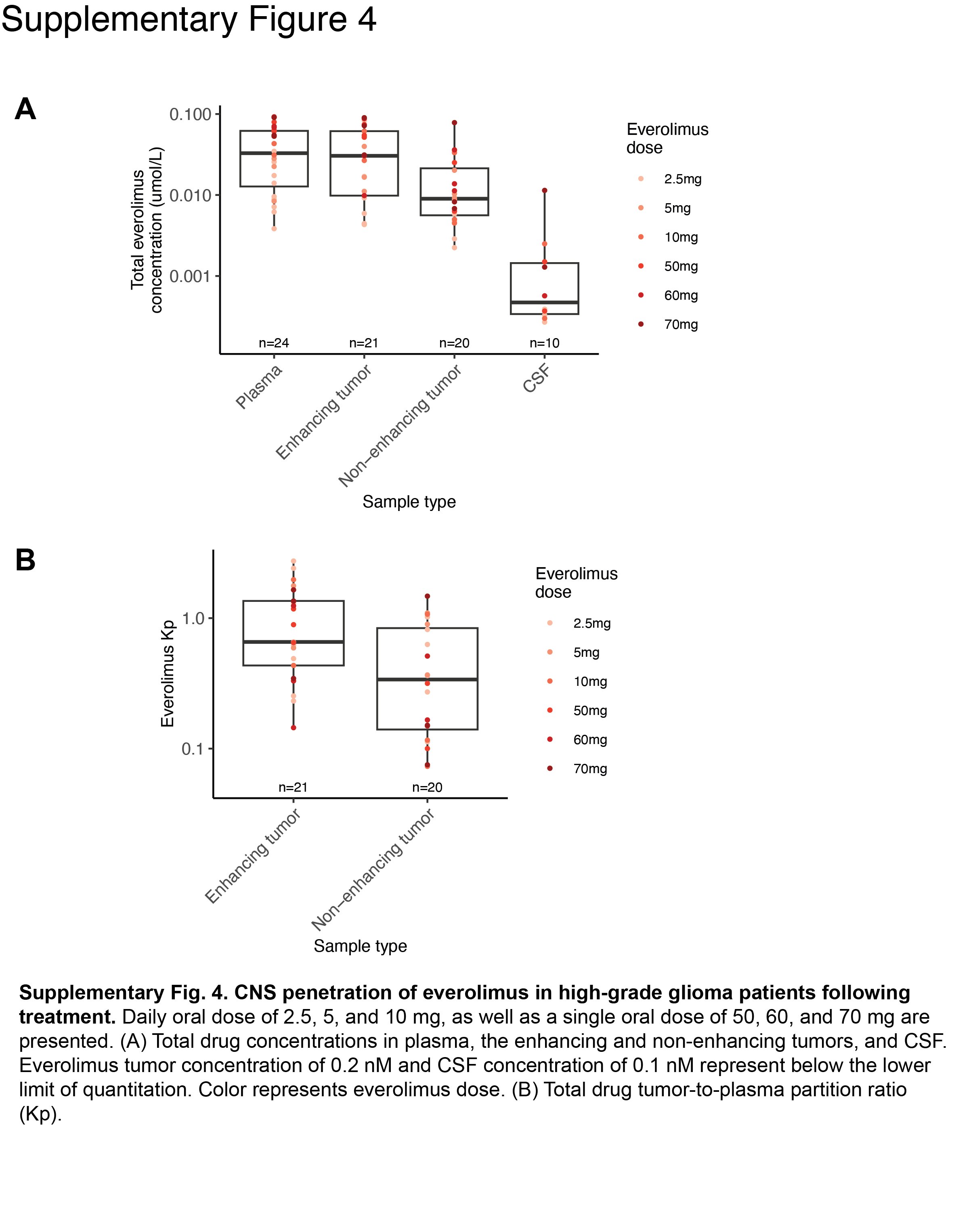

Supplement: noaf257_Supplementary_Data [file noaf257_supplementary_data.zip › noaf257_Supplementary_Data/SupplementaryFigure4.png]

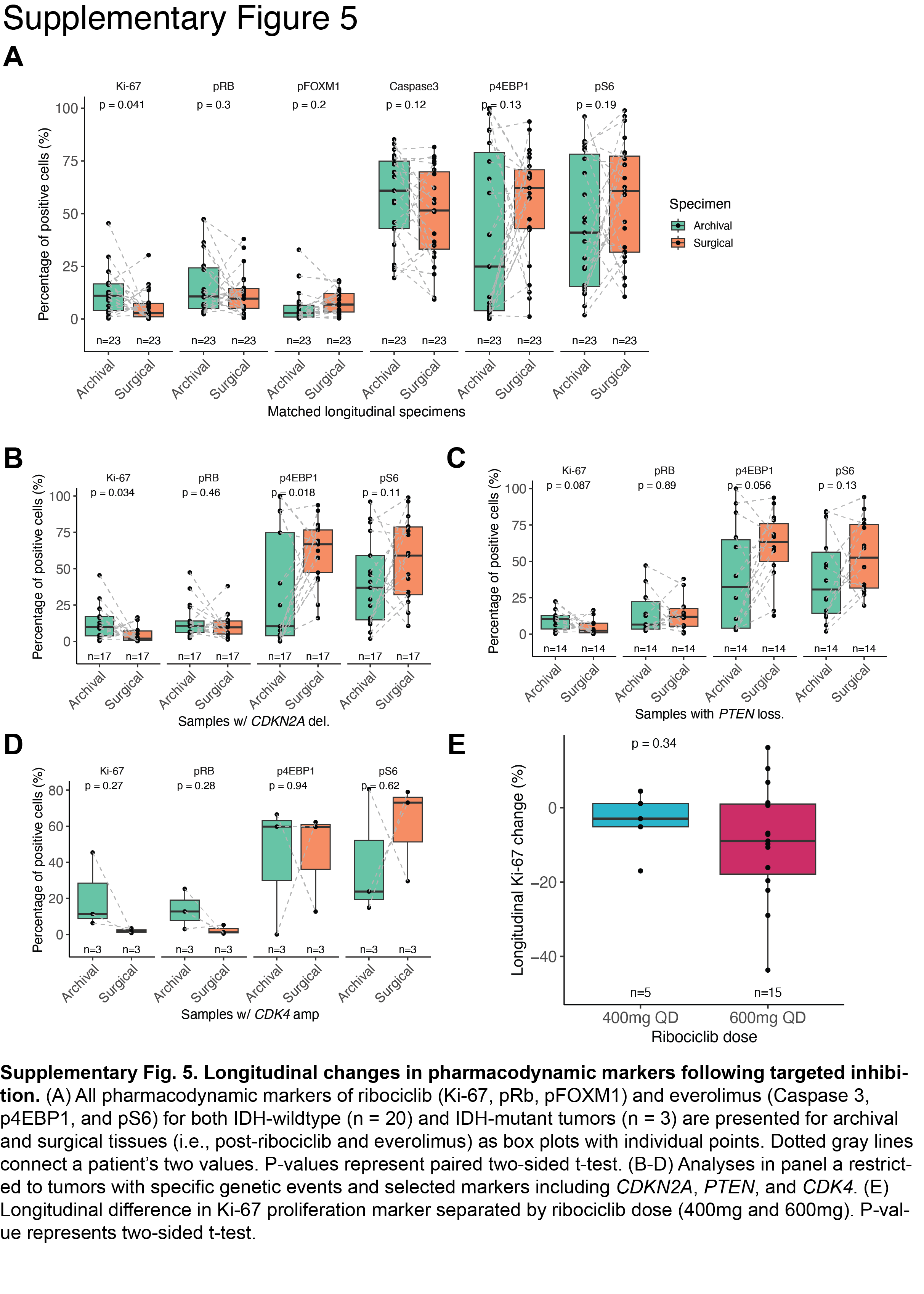

Supplement: noaf257_Supplementary_Data [file noaf257_supplementary_data.zip › noaf257_Supplementary_Data/SupplementaryFigure5.png]

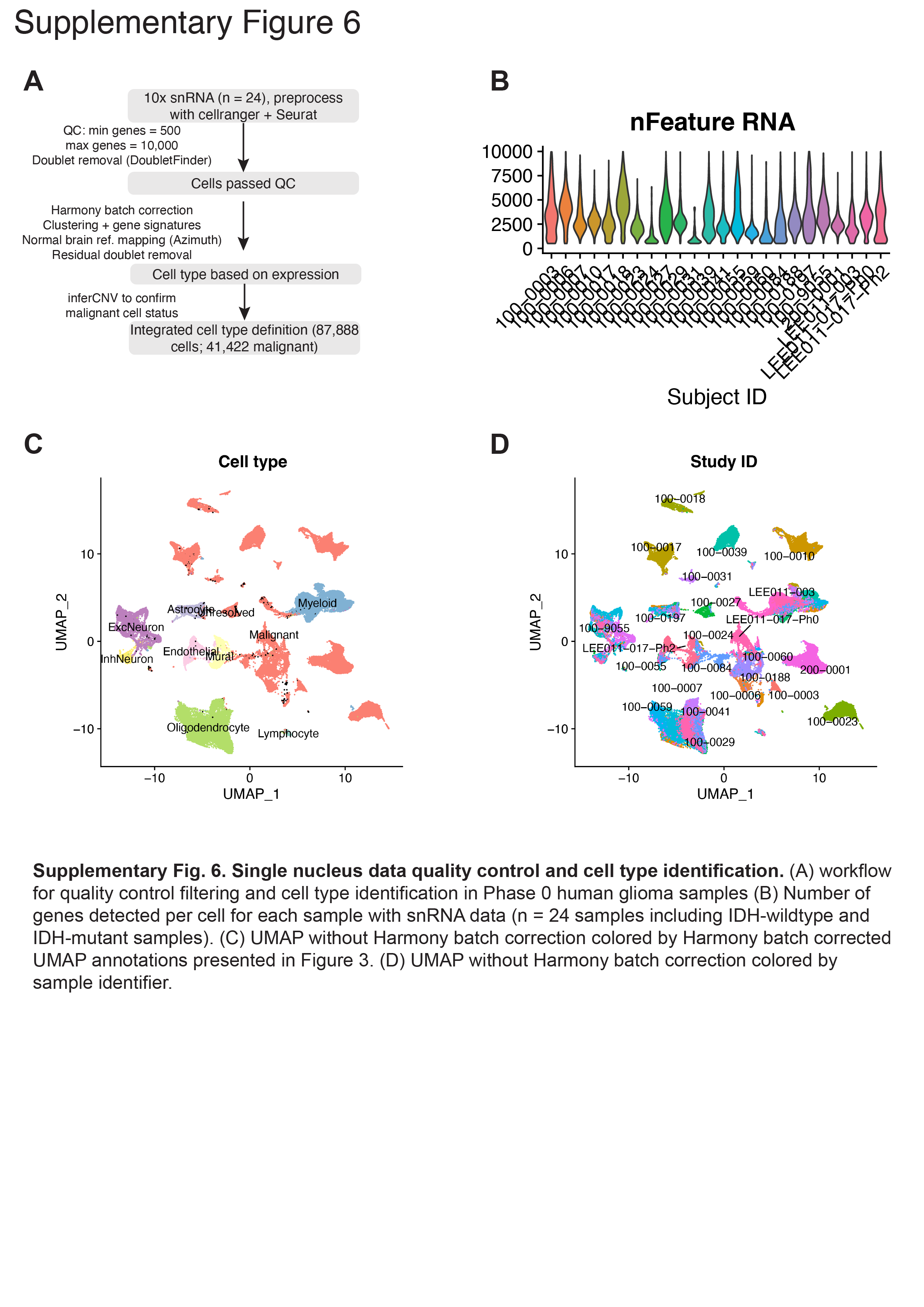

Supplement: noaf257_Supplementary_Data [file noaf257_supplementary_data.zip › noaf257_Supplementary_Data/SupplementaryFigure6.png]

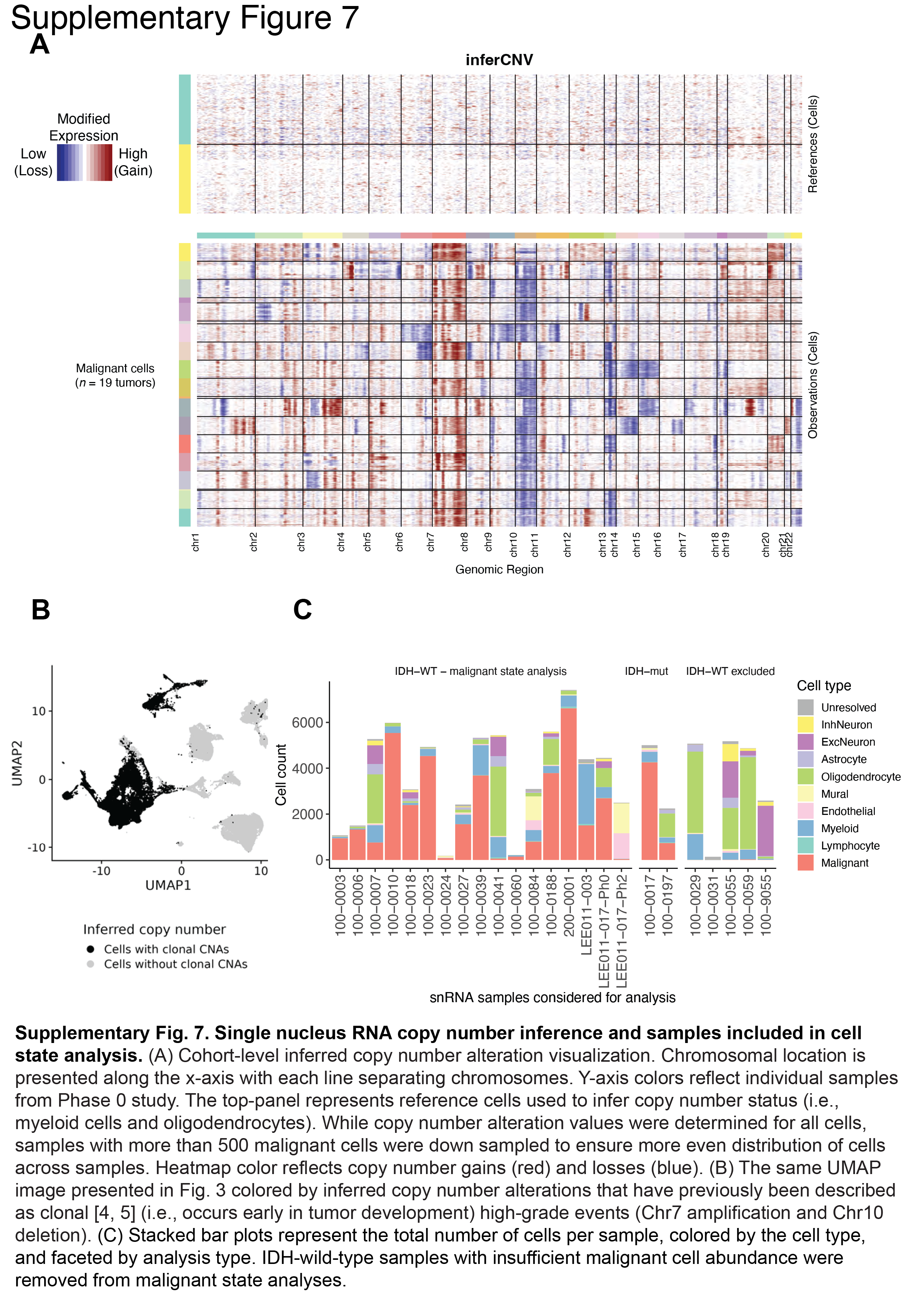

Supplement: noaf257_Supplementary_Data [file noaf257_supplementary_data.zip › noaf257_Supplementary_Data/SupplementaryFigure7.png]

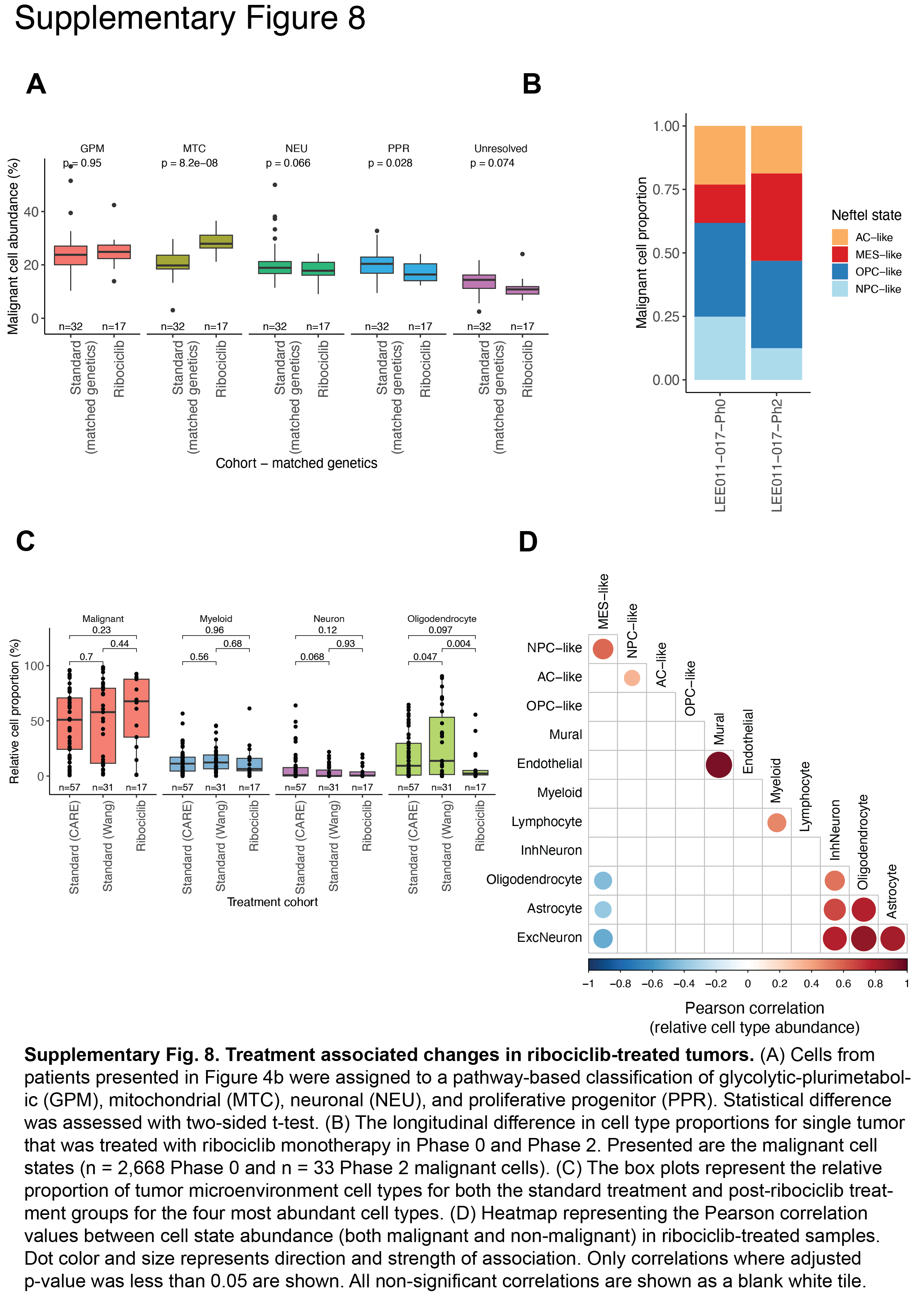

Supplement: noaf257_Supplementary_Data [file noaf257_supplementary_data.zip › noaf257_Supplementary_Data/SupplementaryFigure8.png]

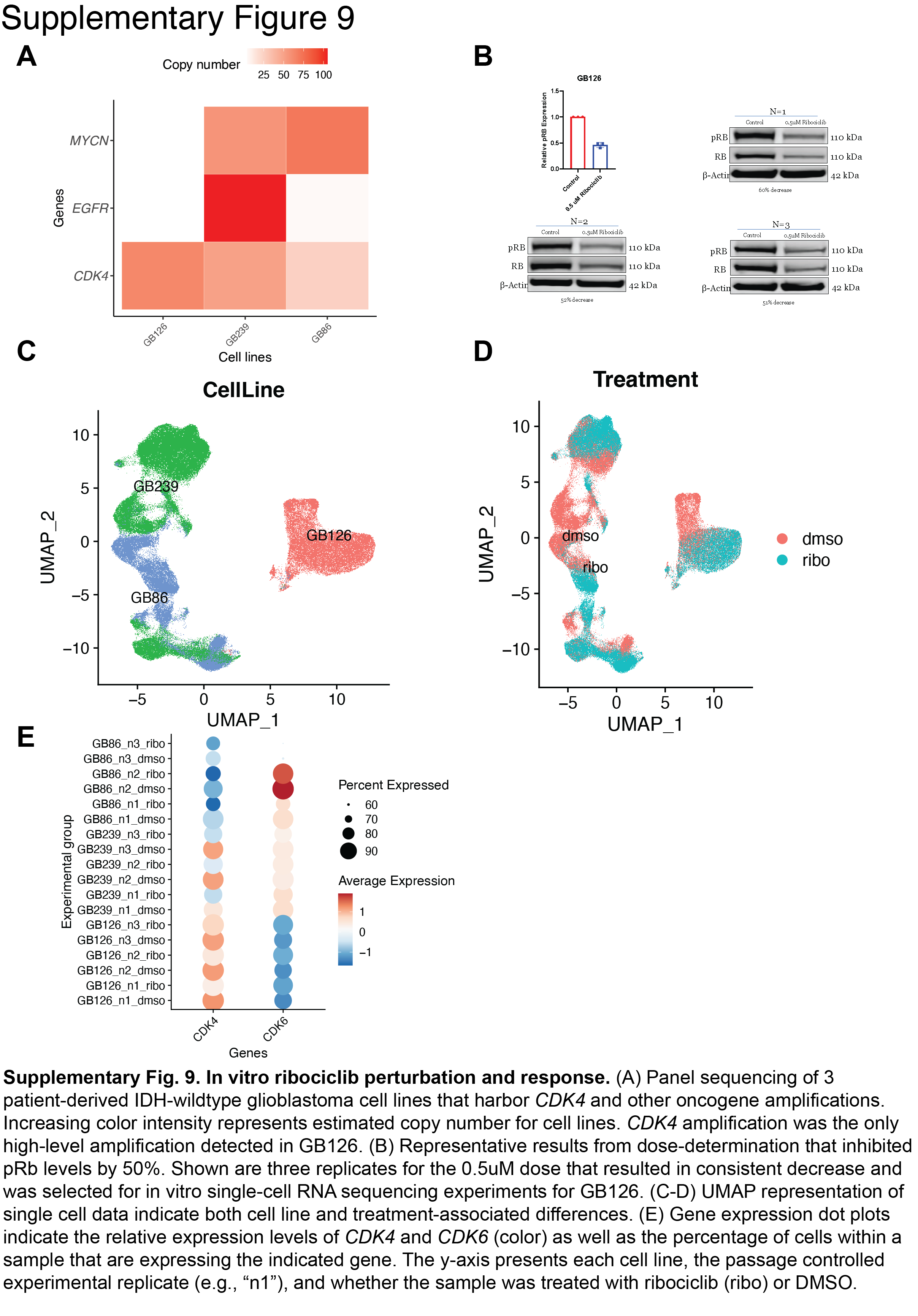

Supplement: noaf257_Supplementary_Data [file noaf257_supplementary_data.zip › noaf257_Supplementary_Data/SupplementaryFigure9.png]
